# Stabilization of the Benzene Radical Trianion in an Inverse‐Sandwich Yttrium Complex

**DOI:** 10.1002/anie.202521849

**Published:** 2025-12-09

**Authors:** Weiqing Mao, Saroshan Deshapriya, Shenglai Yao, Christian Lorent, Matthias Driess, Selvan Demir

**Affiliations:** ^1^ Department of Chemistry: Metalorganics and Inorganic Materials Technische Universität Berlin Strasse des 17. Juni 135, Sekr. C2 10623 Berlin Germany; ^2^ Department of Chemistry Michigan State University 578 South Shaw Lane East Lansing Michigan 48824 USA; ^3^ Department of Chemistry, Physical and Biophysical Chemistry Technische Universität Berlin Strasse des 17. Juni 135, Sekr. PC14 10623 Berlin Germany

**Keywords:** Aromaticity, Density functional theory calculations, EPR spectroscopy, Organic radical anions, Rare‐earth metals

## Abstract

Herein, the first report on the isolated and unambiguously proven benzene radical trianion is presented. This unprecedented radical oxidation state of benzene is stabilized through two trivalent rare earth (RE) metal cations each supported by a bis(guanidinate) scaffold. Specifically, the one‐electron chemical reduction of the neutral inverse‐sandwich yttrium complex [[{(Me_3_Si)_2_NC(N*^i^* Pr)_2_}_2_Y]_2_(*μ*–*ƞ* ^6^:*ƞ* ^6^–C_6_H_6_)] **1**, containing a benzene dianion, with potassium graphite (KC_8_) in the presence of [2.2.2]‐cryptand yielded the title complex [K([2.2.2]‐cryptand)][[{(Me_3_Si)_2_NC(N*^i^* Pr)_2_}_2_Y]_2_(*μ*–*ƞ* ^6^:*ƞ* ^6^–C_6_H_6_^•^)] **2**, featuring a benzene radical trianion. Analyses through single‐crystal X‐ray diffraction, EPR and UV–vis spectroscopy, elucidated its molecular structure and revealed strong [Y^III^–(C_6_H_6_)^3–•^–Y^III^] metal–radical interactions. Although the Y centers remain in the +3 oxidation state, the spin density of the unpaired electron resides primarily on the benzene trianion moiety and extends toward the Y^III^ ions. Density functional theory (DFT) calculations on **2** corroborate this assignment and further suggest weak aromaticity for the benzene radical trianion.

## Graphical abstract

A new type of benzene anion state is born: The unprecedented benzene radical trianion embedded in an inverse‐sandwich yttrium complex was synthesized by a one‐electron chemical reduction of its benzene dianion precursor using potassium graphite in the presence of [2.2.2]‐cryptand. Notably, the yttrium ions remain in the +3 oxidation state and the spin density of unpaired electron resides at the non‐planar benzene radical trianion and induces weak aromaticity.

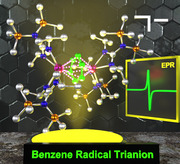

## Introduction

**Chart 1:**
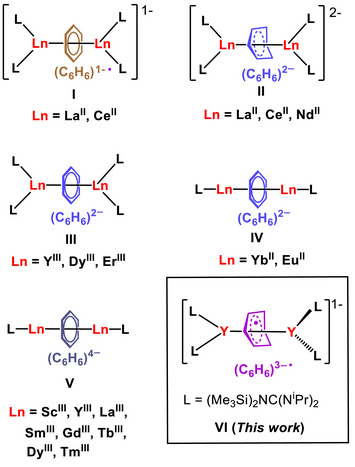
Known inverse‐sandwich RE metal complexes **I**‐**V** containing bridging parent benzene anions, including the first benzene radical trianion complex **VI** of this work, L = monoanionic ligand.

Since the discovery of an inverse‐sandwich benzene complex in 1983,^[^ ^1^ ^]^ species of this class have garnered much interest due to their unique electronic features and bonding characteristics. The symmetric match of the highest occupied molecular orbitals (HOMO) of the reduced benzene ligand and the metal‐based d‐orbitals gives rise to both π and δ interactions. Although the captured benzene is neutral in transition‐metal complexes,^[^ ^1^, ^2^ ^]^ it tends to be reduced to a benzene anion in complexes involving the more electropositive rare earth (RE) metals.^[^ ^3^, ^4^, ^5^, ^6^, ^7^ ^]^ Relative to readily reducible parent polyarenes, such as naphthalene and anthracene, the parent benzene is far more difficult to undergo reduction (−3.42 V versus SCE),^[^ ^8^ ^]^ resulting in fewer RE complexes innate to a reduced benzene, compared to other arenes.^[^ ^4^, ^5^ ^]^ Despite this circumstance and synthetic challenge, benzene has been demonstrated to function as redox‐active bridging ligand due to its ability to accommodate multiple electrons, ranging from formal charges of −1 (7π‐electron benzene radical monoanion), −2 (8π‐electron benzene dianion), to −4 (10π‐electron benzene tetraanion). The first inverse‐sandwich RE complex containing a reduced parent benzene, [K(18‐crown‐6)(*η* ^2^‐C_6_H_6_)_2_][(Cp^tt^_2_La)_2_(*μ*‐*η* ^6^:*η* ^6^‐C_6_H_6_)] (Cp^tt^ = *η* ^5^‐C_5_H_3_(CMe_3_)_2_–1,3), was isolated by the group of Lappert in 1998. In this case, the benzene is reduced to a radical monoanion that is bridging two La^II^ sites as uncovered by single‐crystal X‐ray diffraction (SC‐XRD) and electron paramagnetic resonance (EPR) analyses (Chart 1, I).^[^ ^9^ ^]^ Accordingly, a series of related inverse‐sandwich RE metal complexes, containing mono‐ and dianionic parent benzene, respectively, [K(18‐crown‐6)]*_n_* [(Cp*^X^*_2_Ln)_2_(*μ*‐*η* ^6^:*η* ^6^‐C_6_H_6_)] (*n *= 1 or 2; Cp*^X^ *= Cp″ or Cp′, Cp″ = *η* ^5^‐C_5_H_3_(SiMe_3_)_2,_ Cp′ = *η* ^5^‐C_5_H_4_(SiMe_3_); Ln = La, Ce, Nd) (Chart 1, I‐II), were isolated by the groups of Evans and Roesky, respectively.^[^ ^10^, ^11^, ^12^ ^]^ More recently, the neutral inverse‐sandwich RE^III^ complexes featuring a remarkably planar antiaromatic benzene dianion [{(Me_3_Si)_2_NC(N*^i^* Pr)_2_}_2_RE]_2_(*μ*–*ƞ* ^6^:*ƞ* ^6^–C_6_H_6_) (RE = Y, Dy, Er) were published by the Demir group (Chart 1, III).^[^ ^13^ ^]^ Afterward, Huang and coworkers reported an inverse‐sandwich Eu^II^ complex with the planar parent benzene dianion in a triplet ground state (Chart 1, IV).^[^ ^14^ ^]^ Among the inverse‐sandwich RE metal benzene complexes, those containing a benzene tetraanion have been the subject of extensive studies (Chart 1, V), where their stabilization necessitated superbulky monoanionic ligands on each RE metal center. In fact, the groups of Anker,^[^ ^15^ ^]^ Harder,^[^ ^16^ ^]^ and Huang,^[^ ^17^ ^]^ utilized bulky *β*‐diketiminate ligands to gain access to neutral inverse‐sandwich RE metal complexes containing the benzene tetraanion, [[(BDI)RE(THF)*_n_*]_2_(*μ*‐*η* ^6^:*η* ^6^‐C_6_H_6_)] (BDI = HC[C(Me)N(C_6_H_3_‐R_2_‐2,6)]_2_; R = 3‐pentyl, RE = Y, Sm; R = Cy, Ln = Sm). In comparison, Long and coworkers employed sterically congested pentaisopropylcyclopentadienyl ligands to reach neutral inverse‐sandwich RE metal complexes innate to a benzene tetraanion.^[^ ^18^ ^]^ The substance class of superbulky pentaarylcyclopentadienyls was also employed by Cheng and coworkers to afford analogous La^III^ complexes, [[(Cp^Ar5^)La(THF)*_n_*]_2_(*μ*‐*η* ^6^:*η* ^6^‐C_6_H_6_)] (*n *= 0, 1; Cp^Ar5^ = *η* ^5^‐C_5_Ar_5_, Ar = *^i^* Pr_2_‐C_6_H_3_‐3,5).^[^ ^19^ ^]^ By contrast, bulky amidinate ligands played a key role to isolate amidinate RE complexes, [[(*κ* ^1^:*η* ^6^‐Piso)RE]_2_(*μ*‐*η* ^6^:*η* ^6^‐C_6_H_6_)] (Piso = {(DippN)_2_C*^t^* Bu}, Dipp = C_6_H_3_ *^i^* Pr_2_‐2,6), RE = Y, Gd, Tb, Dy), as disclosed by Winpenny, Mills, and Zheng,^[^ ^20^ ^]^ and a Sc complex, reported by Chu and coworkers,^[^ ^21^ ^]^ each featuring a bridging benzene tetraanion.

Notably, despite the variety of observed anionic oxidation states for benzene in coordination compounds, a parent benzene radical trianion has been never isolated with any metal ion. In light of the overwhelming success of capturing benzene in differing oxidation states with RE metals, we set out to probe the accessibility of the first parent benzene radical trianion with these elements.

We hypothesized that an inverse‐sandwich RE metal complex with a held parent benzene dianion may serve as a platform to reach a trianionic benzene state when exposed to a chemical reductant. In fact, we chose to subject the recently discovered neutral inverse‐sandwich yttrium complex [[{(Me_3_Si)_2_NC(N*^i^* Pr)_2_}_2_Y]_2_(*μ*–*ƞ* ^6^:*ƞ* ^6^–C_6_H_6_)] **1**,^[^ ^13^ ^]^ to a one‐electron chemical reduction benefiting from the diamagnetic nature of both the yttrium(III) ions and the bridging benzene dianion. Herein, we report the first example of a fully characterized metal complex **2**, [K([2.2.2]‐cryptand)][[{(Me_3_Si)_2_NC(N*^i^* Pr)_2_}_2_Y]_2_(*μ*–*ƞ* ^6^:*ƞ* ^6^–C_6_H_6_^•^)] containing a benzene radical trianion (Chart 1, VI), including its solid‐state molecular structure.

## Results and Discussion

**Scheme 1:**
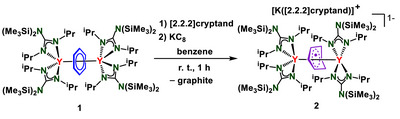
Synthesis of [K([2.2.2]‐cryptand)][[{(Me_3_Si)_2_NC(N*^i^* Pr)_2_}_2_Y]_2_(*μ*–*ƞ* ^6^:*ƞ* ^6^–C_6_H_6_^•^)], **2** through chemical reduction of **1** with KC_8_ in the presence of [2.2.2]‐cryptand.

The controlled one‐electron reduction of **1** with KC_8_ in the presence of [2.2.2]‐cryptand proceeded in benzene, and was accompanied by a rapid color change from orange‐yellow to orange‐brown. Orange‐brown crystalline solids of **2** were obtained from the resulting mixture in 86% yield after recrystallization (Scheme 1).

**Figure 1:**
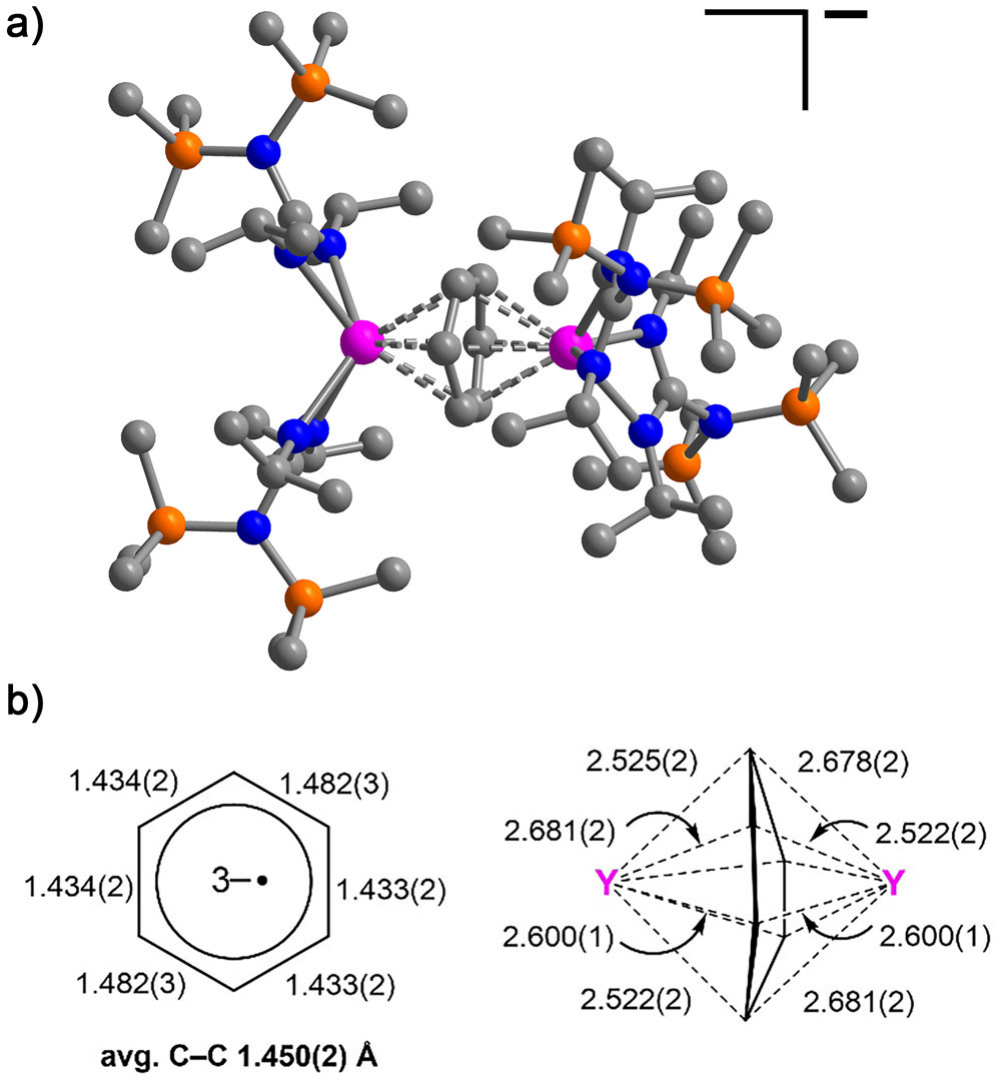
a) Molecular structure of the anion of [K([2.2.2]‐cryptand)][[{(Me_3_Si)_2_NC(N*^i^* Pr)_2_}_2_Y]_2_(*μ*–*ƞ* ^6^:*ƞ* ^6^–C_6_H_6_^•^)], **2**. Pink, orange, blue, and gray spheres represent Y, Si, N, and C atoms, respectively. All hydrogen atoms, solvent molecules, and the counterion [K([2.2.2]‐cryptand)]^+^ are omitted for clarity; b) Schematic representation of selected distances (Å) and structural features of the benzene radical trianion bound to Y^III^ in **2**.

Dark orange‐brown crystals of **2** suitable for a SC‐XRD analysis were grown by slow evaporation of a saturated hexane solution at room temperature. Compound **2** is an ion pair consisting of the [[{(Me_3_Si)_2_NC(N*^i^* Pr)_2_}_2_Y]_2_(*μ*–*ƞ* ^6^:*ƞ* ^6^–C_6_H_6_^•^)]^−^ complex anion (Figure 1a) and the [K([2.2.2]‐cryptand)]^+^ counter cation. The crystallographic analysis revealed significant structural changes of the benzene moiety traversing from **1** to **2** (Table 1). In **2**, the benzene ring is distorted from planarity and exhibits a boat conformation with the largest C─C─C─C torsion angle of 16.6°, while the benzene ring in **1** is essentially planar with a tiny C─C─C─C torsion angle of 0.2° (Figure 1b and Table 1). To note, ligated parent benzene can exhibit distortions from planarity, as was observed in some inverse‐sandwich RE metal complexes with benzene in the di‐ or tetraanionic state.^[^ ^11^, ^16^, ^17^ ^]^ Recent studies have shown that the planarity of metal–benzene ligands depends not only on the benzene charge state but also on the metal's size and covalency. It has been demonstrated that greater M─C covalency and smaller metal ionic radii lead to stronger metal–benzene interactions and increased ring puckering in alkaline‐earth complexes.^[^ ^22^ ^]^ Accordingly, the relatively small and covalent Y^III^ center in complex **2** is expected to induce a boat‐like distortion of the benzene trianion. Similar non‐planar benzene ligands have been reported in related Y–benzene complexes,^[^ ^17^ ^]^ consistent with our structural observations. The C─C distances of the coordinated benzene ring in **2** marginally vary between 1.433(2) and 1.482(3) Å, (Figure 1b, Table 1), but are substantially longer than those of the parent “free” benzene (1.3913(1) Å),^[^ ^23^ ^]^ suggesting that the negative charges are evenly distributed among the six carbon atoms. This is different from the quinoidal structure of the benzene 1,4‐dianion in **1** with two‐short and four‐long C─C distances in several RE and alkaline‐earth metal benzene complexes.^[^ ^13^, ^14^, ^16^, ^24^, ^25^, ^26^, ^27^, ^28^ ^]^ Remarkably, the one‐electron reduction of **1** to **2** is accompanied by a significant shortening of the intermetallic Y⋯Y distance from 4.515(1) in **1** to 4.324(1) Å in **2**. Another striking structural difference is an approximately 71° rotation of one bis(guanidinate) yttrium scaffold with respect to the other (the rotation angle is defined as the C(backbone)‐Y‐Y‐C(backbone) torsion angle). The shorter Y⋯Y distance originates from a stronger metal‐benzene interaction owing to a higher negative charge, which, in turn, increases the steric repulsion between the opposing guanidinate ligands forcing them to rotate. The average Y─N_guan_ distance in **2** (2.458(2) Å) is slightly longer (ca. 0.07 Å) than that in **1** (2.384(2) Å; Table 1). The slight Y─N_guan_ elongation of the distance suggests a weakening of the RE─N bonds due to the increased electrostatic attraction between the Y ions and the benzene trianion, as is evident by the shorter interatomic Y─C_arene_ distances (2.522(2) to 2.681(2) Å in **2** versus 2.662(2) to 2.702(2) Å in **1**; Table 1) and the average Y─C_cent_ distances (2.162(5) Å in **2** versus 2.268(4) Å in **1**, C_cent_ = the ring centroid of the bound benzene moiety). In addition, steric repulsion between the opposing guanidinate ligands upon reduction may further contribute to the observed elongation of the Y─N_guan_ bonds. The relatively similar average of the Y─N_guan_ interatomic distances in **1** and of the anion in **2** hint at the oxidation state of +3 for the Y ions in **2**. This assignment takes into account that an oxidation state of +2 would correspond to an increase in ionic radius by nearly 0.1 Å.^[^ ^29^ ^]^ Totaling all structural features alludes to a [Y^III^─(C_6_H_6_)^3–•^─Y^III^] motif for the electronic structure of the anion in **2**. This assignment is confirmed by the results gained through EPR spectroscopy and density functional theory (DFT) calculations, as discussed below.

**Table 1:** Selected distances (Å) and torsion angles (°) of the benzene dianion ligand in **1** ^13^ versus the benzene radical trianion ligand in **2**. The DFT‐calculated metrics for the benzene radical trianion ligand in **2** were obtained at the uTPSSh/def2‐TZVP level of theory [in brackets].

	1	2
*d* (C–C)	1.372(3)	1.434(2) [1.436]
	1.451(1)	1.434(2) [1.435]
	1.490(3)	1.482(3) [1.481]
	1.372(3)	1.433(2) [1.436]
	1.451(1)	1.433(2) [1.436]
	1.490(3)	1.482(3) [1.481]
av *d* (C─C)	1.438(2)	1.450(2) [1.451]
*d* (Y1…Y2)	4.515(1)	4.324(1) [4.305]
*d* (Y─C_arene_)	2.662(2)–2.702(2)	2.522(2)–2.681(2) [2.516–2.699]
av *d* (Y─C_cent_)	2.268(4)	2.162(5) [2.172]
av *d* (Y─N_guan_)	2.384(2)	2.458(2) [2.457]
C─C─C─C	0.2	16.6 [16.3]

**Figure 2:**
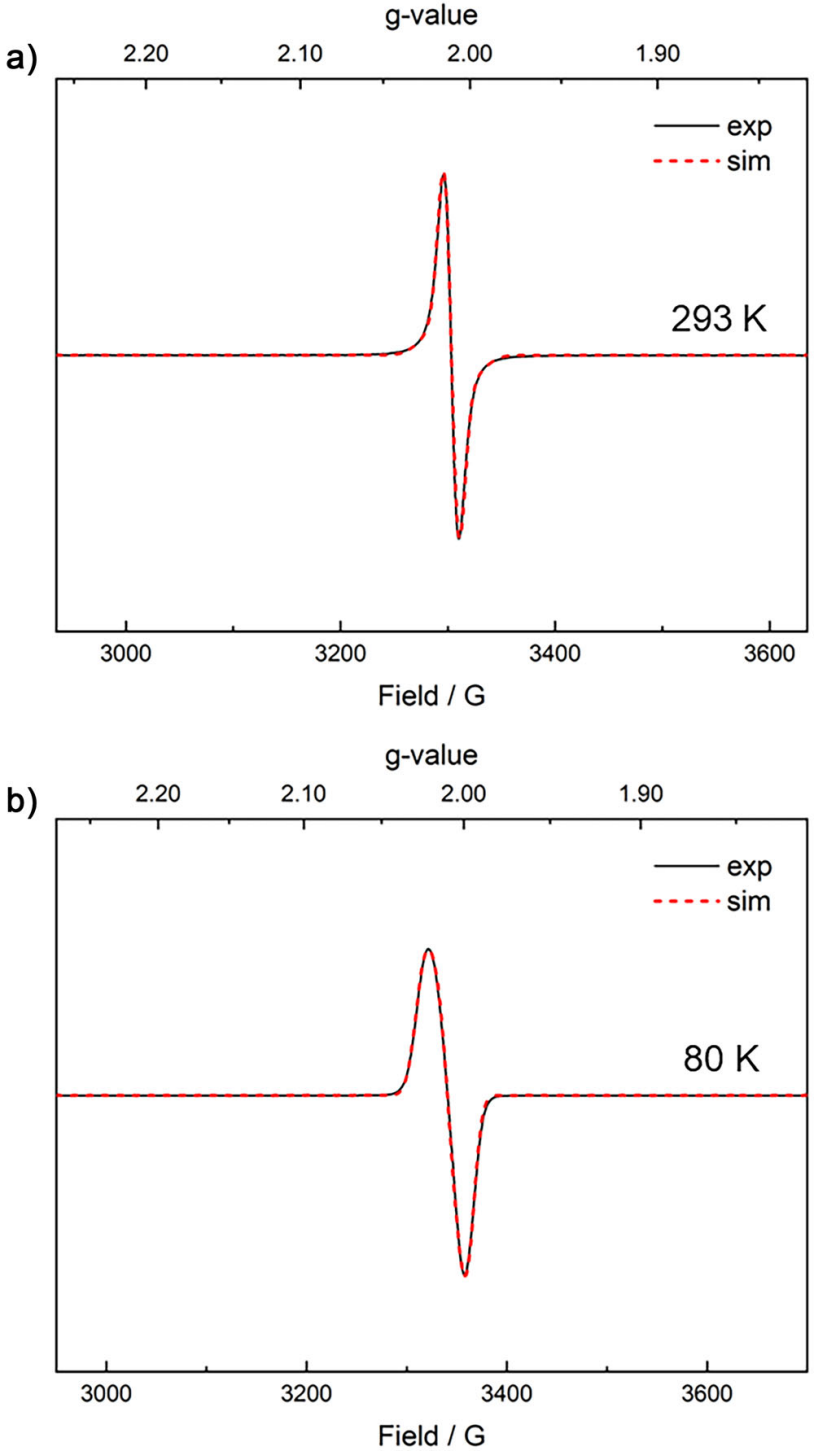
a) CW X‐band EPR spectra of **2** recorded in benzene at 293 K; b) CW X‐band EPR spectra of **2**, recorded in benzene at 80 K. Experimental conditions: microwave frequency *ν* = 9.3–9.4 GHz, microwave power = 1 mW, modulation amplitude = 2 G, and modulation frequency = 100 kHz. Experimental and simulated spectra are shown as solid black and dashed red traces, respectively.

EPR spectroscopy was applied to verify the paramagnetic nature of the benzene radical trianion. The EPR spectrum of **2**, measured at 293 K (Figures 2a and S3), displays an isotropic, sharp signal at *g* = 2.011 (line width = 14 G). This signal type and *g* value is typical for organic radicals, but does not feature characteristic ^89^Y hyperfine splitting, indicative of the unpaired electron not being located at the Y center. Similarly, the EPR spectra at 80 K (Figures 2b and S4), also exhibit an isotropic, somewhat broader signal at *g* = 2.011 (line width = 36 G) which lacks ^89^Y‐hyperfine coupling features. In addition, no hyperfine coupling with the ^1^H nuclei was observed. This could originate from increased line broadening due to the presence of heterogeneous microstates, related to structural dynamicity, which is suggested from the increased *g*‐strain (Table S1) and/or solvent effects.^[^ ^30^ ^]^ The power saturation curve (Figure S5) exhibits no indications for dipolar coupling, in line with only one spin located at the benzene ring.

DFT calculations were carried out to uncover the electronic structure of the anion in **2**. Crystal coordinates of the anionic part of **2** served as the starting point for the DFT geometry optimization. The optimized coordinates were then employed in the subsequent DFT calculations. Upon optimization, the benzene ring retains its distorted geometry where the distortion stems from not only the presence of the 3– charge on the benzene scaffold but its interactions with the Y^III^ ions, in agreement with the experimental structure. This was confirmed by the geometry optimization of an uncoordinated benzene trianion resulting in a planar cyclohexanedienediide type structure (Figure S8).

**Figure 3:**
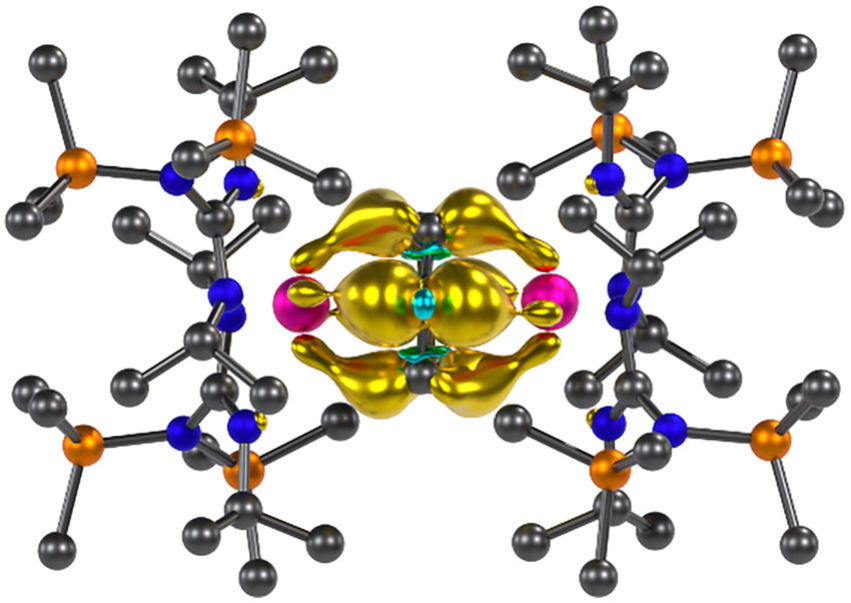
DFT‐derived spin‐density distribution in the anion of **2**. Pink, orange, blue, and gray spheres represent Y, Si, N, and C atoms, respectively. Gold and teal surfaces represent different phases of spin density. H atoms and counter cation [K([2.2.2]‐cryptand)]^+^ have been omitted for clarity. Isovalue set at 0.003 for all surfaces.

The spin density of the paramagnetic anion in **2** illustrates the distribution of the unpaired electron (Figure 3). The spin primarily resides on the bridging benzene and extends toward the Y^III^ centers, which is consistent with the natural spin population values. Only marginal spin density is found on the guanidinate scaffold in accordance with the assignment of a benzene‐centered radical (Table S5). In addition, a modified localized orbital bonding analysis (mLOBA)^[^ ^31^ ^]^ yielded +3 oxidation states for the yttrium centers and an overall −3 state for the bridging benzene ligand, further confirming the trianionic benzene state (Table S5). This method assigns integer oxidation states based on the electron populations of localized molecular orbitals. Unlike natural population analysis, which yields fractional electron populations and may not map directly to integer oxidation states, mLOBA analyzes the contributions of localized occupied orbitals to determine electron ownership consistent with the IUPAC definition.

The bond distances within the optimized structure of **2** are scrutinized against those extracted from single‐crystal X‐ray diffraction analysis. The DFT‐calculated metrics for **2** (see Table 1) coincide well with the experimental bond distances which emphasizes that the used DFT methodologies lead to a good representation of this system. Natural bond orbital (NBO) theory analysis was performed on the structure of **2** following its geometry optimization to determine the bonding interactions of the bridging benzene motif and the guanidinate scaffold with the yttrium centers. According to the second‐order perturbation analysis, the Y─C_arene_ interactions are of predominantly ionic character where the donor orbitals are lone pairs on the C atoms and the C─C bonding orbitals. No apparent back donation is predicted from the yttrium centers towards the arene ring. Similarly, all prominent Y–guanidinate interactions are of ionic nature and arise from the lone pairs on the coordinating N atoms and the N─C bonding orbitals. This type of ionic bonding picture is somewhat akin to that reported for **1**.^[^ ^13^ ^]^

**Figure 4:**
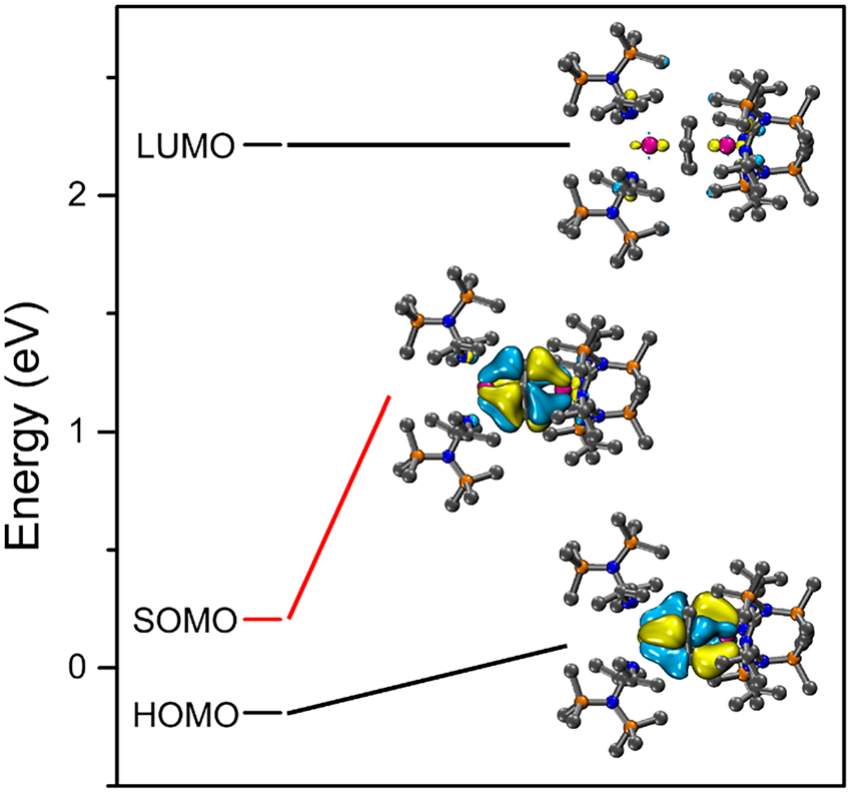
Frontier molecular orbitals of **2** calculated using a uTPSSh functional at the def2‐TZVP level (see Supporting Information for details). Pink, orange, blue, and gray spheres represent Y, Si, N, and C atoms, respectively. H atoms are omitted for clarity. Isovalue for all surfaces is 0.03.

The highest occupied molecular orbital (HOMO) stems solely from bonding interactions between the sandwiched benzene ring and the Y centers, Figure 4. The orbital distribution is described as a δ bond between the 4*d* orbitals of the Y^III^ centers and the π* orbitals of the benzene bridge. The singly occupied molecular orbital (SOMO) features some phase changes but overall exhibits a similar orbital distribution to the HOMO and is congruent with the distribution of the spin density displayed in Figure 3. By contrast, the composition of the lowest unoccupied molecular orbital (LUMO) is mainly of 4*d* character of Y^III^ and features minor contributions from all guanidinate ligands. The relative energies and the distribution of the frontier orbitals are illustrated in Figure 4.

**Figure 5:**
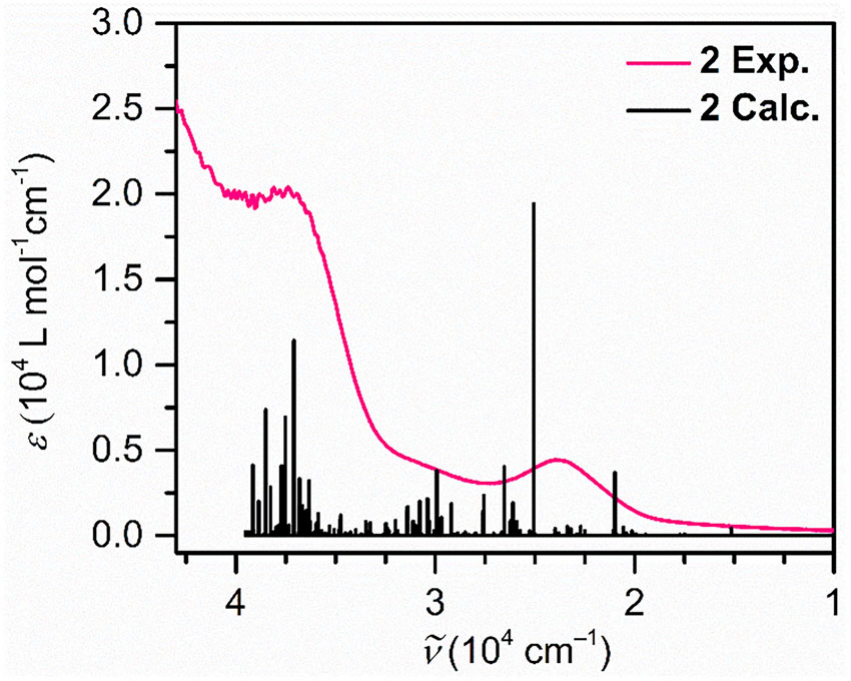
UV–vis spectrum of **2** collected in Et_2_O. Experimental spectrum (pink trace) was collected on a sample with 100 µM concentration. TD‐DFT predicted transitions are shown by black vertical lines.

The UV–vis spectrum of **2** was collected on a 100 µM analyte solution in Et_2_O. The spectrum shows absorption in the UV region and throughout the visible spectrum (Figure 5). Three main absorption features are centered around 268 nm (3.73 × 10^4^ cm^−1^), 335 nm (2.99 × 10^4^ cm^−1^), and 422 nm (2.37 × 10^4^ cm^−1^). The feature at 422 nm is broad and exhibits absorption toward the end of the visible region. More intense absorption toward the lower wavelengths is in accordance with the orange‐brown color of the solution. The lack of new absorption features toward higher wavelengths implies that there are no electronic excitations stemming from the Y ions, consistent with the yttrium centers being assigned as trivalent. The individual electronic excitations giving rise to the UV–vis absorption features were uncovered via TD‐DFT calculations performed on the optimized coordinates of the anion in **2** with a Et_2_O solvent model (Figure 5). The strongest predicted absorption appears at 399 nm (2.51 × 10^4^ cm^−1^) and is attributed to a ligand‐to‐metal charge transfer (LMCT) from the HOMO into molecular orbitals primarily of Y^III^ 4*d* character, with minor contributions from the guanidinate ligands.

The second most intense excitation found at 270 nm (3.71 × 10^4^ cm^−1^) corresponds to another LMCT, consisting of excitations from HOMO and SOMO to primarily yttrium 4*d*‐based MOs. In the UV region, at 260 nm (3.85 × 10^4^ cm^−1^), the next most intense absorption is predicted which, however, mainly consists of ligand‐to‐ligand charge transfers (LLCTs) arising from HOMO and SOMO to guanidinate‐based MOs. The absence of any metal‐to‐metal or metal‐to‐ligand charge transfer bands confirm that the Y centers remain diamagnetic upon chemical reduction, and hence are in the +3 oxidation state. Detailed information about the most intense TD‐DFT transitions is listed in Table S4 of the Supporting Information.

**Figure 6:**
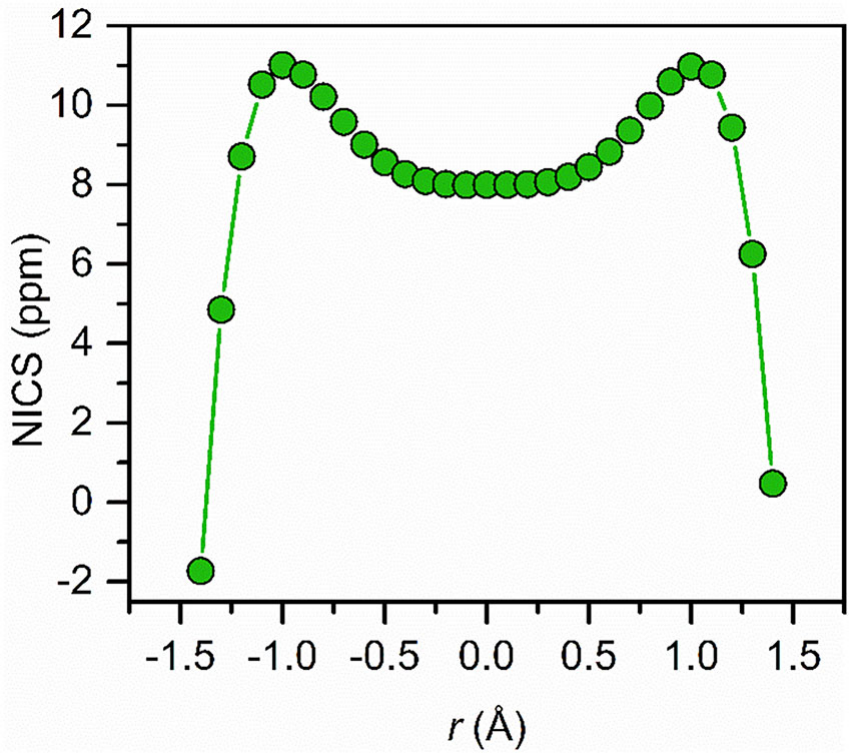
Nucleus‐independent chemical shift (NICS) values calculated for points along the axis perpendicular to the bridging benzene ring of the anion in **2**.

Nucleus‐independent chemical shift (NICS) values were calculated along an axis perpendicular to the benzene ring of the radical trianion in **2** to probe its aromaticity via magnetic shielding tensors (Figure 6).^[^ ^32^ ^]^ The NICS values were generated by taking the negative value of the isotropic chemical shielding magnitudes at dummy atoms defined along the axis. NICS values follow a trend with a NICS(0) value of 7.99 ppm at the centroid and then a gradual increase to a NICS(1) value of about 11 ppm, 1 Å on either direction of the centroid. Beyond 1 Å, a sharp decrease is observed where the values fall below 0 closer to ±1.5 Å. Notably, the arising trend of the NICS values as reflected by the two maxima in Figure 6, is quite exceptional. Intriguingly, at first glance, the system appears to be antiaromatic, owing to the positive NICS values which typically indicate a paratropic ring current, suggestive of antiaromaticity. However, the interpretation is more intricate in this case, as the unpaired electron on the benzene ring may generate a paratropic ring current owing to its paramagnetic nature. As a result, the system may appear antiaromatic but might have in reality some degree of aromatic character. Thus, we conducted further studies to gain more insight, as outlined below. However, it should be noted that there is precedence of exploring aromaticity of paramagnetic species via a NICS analysis, which gives our study validity.^[^ ^33^ ^]^

We proceeded with a comparative study where the NICS values of **2** were compared to those of **1**.^[^ ^13^ ^]^ The relevant data of **1** follow a trend with a maximum positive NICS value at the centroid and then a gradual decline on either side of the ring, suggestive of antiaromaticity. Even though the NICS data of **2** follow this general trend, Figure 6, a dip is observed in NICS values when moving from ±1 to 0 Å. Since **1** is assigned as antiaromatic, the observed dip for **2** may suggest that the π‐electron system of 4n + 1 lowers antiaromaticity, especially near the ring centroid. Noteworthy, the calculated NICS(0) and NICS(1) values for **1** are about 5 to 8 times higher than those for **2**, signifying **2** to be of less antiaromatic nature relative to **1**.

For comparison, the NICS values were calculated for the hypothetical complex [[{(Me_3_Si)_2_NC(N*^i^* Pr)_2_}_2_Y]_2_(*μ*–*ƞ* ^6^:*ƞ* ^6^–C_6_H_6_)]^2–^, bearing a bridging tetraanionic benzene ligand. Here, the calculations of the NICS values followed the same procedure as for **2** and afforded negative values, signifying aromaticity (Figure S9). Hence, deploying more electrons onto the benzene ring changes the nature of the arene bridge in that it traverses from antiaromaticity to aromaticity. Specifically, the benzene dianion in **1** is antiaromatic, the benzene trianion in **2** is weakly aromatic, and the benzene tetraaanion in the hypothetical compound is aromatic.

Building on our interpretation of the NICS data for **2** on hand and appreciating the complexity brought on by the unpaired electron, a Harmonic oscillator model of aromaticity (HOMA) was considered next.^[^ ^34^, ^35^ ^]^ This model evaluates the geometry of the arene system and takes into account the deviation from the geometry of neutral benzene to predict aromaticity. A system that is unambiguously aromatic has a HOMA value of +1, a non‐aromatic system has a value of 0 and an antiaromatic system produces a significantly negative value (often around −0.5). Importantly, in the case of **2**, the HOMA model will be unaffected by the unpaired electron. In fact, this model has been successfully employed for the interpretation of non‐planar and/or paramagnetic systems.^[^ ^36^ ^]^

The HOMA value calculated for the bridging benzene moiety of **2** is −0.135, Table S6. Albeit a negative value, its small magnitude does not strongly support an antiaromaticity assignment. Therefore, the reparametrized HOMA values, HOMAc, were calculated next.^[^ ^37^ ^]^ The calculations via this recently developed HOMAc model provide more accurate estimates regarding antiaromaticity compared to HOMA. Accordingly, a calculated value close to +1 indicates aromaticity, non‐aromaticity is reflected with a value 0 and a value near −1 suggests antiaromaticity. Hence, running this calculation for **2** yielded a HOMAc value of 0.399, Table S7. Consequently, **2** is ascribed to be a system innate to a weak aromatic character.

## Conclusions and Outlook

In summary, we synthesized and characterized the first inverse‐sandwich metal complex [K([2.2.2]‐cryptand)][[{(Me_3_Si)_2_NC(N*^i^* Pr)_2_}_2_Y]_2_(*μ*–*ƞ* ^6^:*ƞ* ^6^–C_6_H_6_^•^)], **2**, containing the unprecedented benzene radical trianion bound to two metal centers. Structural, EPR and UV–vis spectroscopic data provide evidence that the radical anion in **2** features a [Y^III^─(C_6_H_6_)^3–•^─Y^III^] electronic structure with strong yttrium(III)─benzene trianion interactions. Congruently, DFT calculations reveal that significant spin density of the unpaired electron resides at the benzene trianion ring and that strong δ bonding interactions between the Y^III^ ions and the π‐system of the benzene radical trianion exist. The computational results further suggest that the bridging benzene unit of **2** exhibits weak aromatic character. The investigation of the reactivity of **2** and the synthesis and characterization of analogous inverse‐sandwich complexes containing other RE metals are currently in progress.

## Supporting Information

CCDC 2492120 (compound 2) contains the supplementary crystallographic data for this paper. The data can be obtained free of charge via www.ccdc.cam.ac.uk/data_request/cif, or by emailing data_request@ccdc.cam.ac.uk, or by contacting The Cambridge Crystallographic Data Centre, 12 Union Road, Cambridge CB2 1EZ, UK; fax: +44 1223 336033. The authors have cited additional references within the Supporting Information.^[^ ^38^, ^39^, ^40^, ^41^, ^42^, ^43^, ^44^, ^45^, ^46^, ^47^, ^48^, ^49^, ^50^, ^51^, ^52^, ^53^, ^54^, ^55^, ^56^, ^57^ ^]^

## Author Contributions

Selvan Demir and Matthias Driess planned and supervised the investigation. The experimental work was performed by Weiqing Mao (synthesis, elemental analysis, spectroscopic characterization), Shenglai Yao (sc‐XRD), and Christian Lorent (EPR). Saroshan Deshapriya performed all theoretical calculations. The TOC graphical abstract was produced by Saroshan Deshapriya and Selvan Demir. The manuscript was written through the contributions of all authors. All authors have approved the final version of the manuscript.

## Conflict of Interests

The authors declare no conflict of interest.

## Supporting information



Supporting Information

## Data Availability

The data that support the findings of this study are available, in the Supporting Information of this article.
