# Supplementary material for: Stabilization of the Benzene Radical Trianion in an Inverse‐Sandwich Yttrium Complex
Source: Angew Chem Int Ed Engl. 2025 Dec 9;65(13):e21849. doi: 10.1002/anie.202521849 (PMC13007589; doi:10.1002/anie.202521849)
Supplement: Supplementary file 1 — Supporting Information [file ANIE-65-e21849-s001.pdf]

Supporting Information

Stabilization of the Benzene Radical Trianion in an Inverse-Sandwich Yttrium Complex

Weiqing Mao,[a] Saroshan Deshapriya,[b] Shenglai Yao,[a] Christian Lorent,[c] Matthias Driess,*,[a] and Selvan Demir*,[b]

[a]Department of Chemistry: Metalorganics and Inorganic Materials, Technische Universität Berlin, Strasse des 17. Juni 135, Sekr. C2 10623, Berlin, Germany

[b]Department of Chemistry, Michigan State University, 578 South Shaw Lane, East Lansing, Michigan 48824, USA

[c]Department of Chemistry, Physical and Biophysical Chemistry, Technische Universität Berlin, Strasse des 17. Juni 135, Sekr. PC14 10623, Berlin, Germany

Correspondence to: matthias.driess@tu-berlin.de; sdemir@chemistry.msu.edu

Table of Contents

A. Experimental Section……S2

A1 General Considerations……S2

A2 Single-Crystal X-ray Structure Determination……S2

A3 EPR Measurement……S2

A4 Synthesis and Characterization……S3

A5 Details of the Single-Crystal X-ray Diffraction Analysis……S8

B. Computational Section……S10

B1 TD-DFT Calculation……S10

B2 Bond Metrics, Natural Charges, and Oxidation States……S12

B3 HOMA and HOMAc Values……S13

B4 Optimized Coordinates……S14

B5 Calculations on Hypothetical Molecules……S18

C. References……S19

A. Experimental Section

A1. General Considerations

All experiments were carried out under dry, oxygen-free nitrogen using standard Schlenk techniques or an MBraun argon-filled glovebox fitted with a gas purification and recirculation unit. Solvents were dried by standard methods and freshly distilled prior to use. The starting material, $[[\{(Me_3Si)_2NC(N^iPr)_2\}_2Y]_2(\mu{-}\eta^6{:}\eta^6{-}C_6H_6)]$ (**1**)[1] was prepared according to previous work by the Demir group. [2.2.2]-cryptand was purchased from Sigma-Aldrich and used as received. Potassium graphite (KC_8) was prepared by reacting potassium with previously dried graphite in a 1:8 ratio at 160 °C for 2 h under dry nitrogen. Elemental analyses were performed by the analytical labor service in the Institute of Chemistry, Technical University of Berlin, Germany. IR spectra were measured with a Nicolet iS5 FT-IR-Spectrometer from the company Thermo Scientific. UV-vis spectra were recorded on an Analytik Jena Specord S600 diode array spectrometer.

A2. Single-Crystal X-ray Structure Determination

The crystals were mounted on a glass capillary in grease (Apiezon N) and measured in a cold N_2 flow. The data of compound **2** was collected on an Oxford Diffraction Supernova, Single source at offset, Atlas at 150 K (Cu-Kα-radiation, λ = 1.5418 Å). The structure was solved with the SHELXT[2] and refined with Olex2[3-4] software package. The positions of the H atoms were calculated and considered isotropically according to a riding model. Compound **2** crystallized with one molecule of *n*-hexane, which is severely disordered and has been removed with the solvent-mask procedure in Olex2.[3-4] CCDC 2492120 (compound **2**) contains the supplementary crystallographic data for this paper. The data can be obtained free of charge by contacting The Cambridge Crystallographic Data Centre, 12, Union Road, Cambridge CB2 1EZ, UK; fax: +44 1223 336033.

A3. EPR Measurement

The EPR data of compound **2** dissolved in benzene (5 mM) was measured at 293 K and 80 K. The room temperature spectra and the power saturation data were recorded on a Bruker EMXplus spectrometer equipped with an ER 4122 SHQE resonator and an Oxford ESR900 helium flow cryostat controlled by an Oxford ITC4 device. EPR spectra of the frozen solution and crystalline solids were measured with a laboratory-built X-Band spectrometer equipped with a Bruker SHQ resonator, an ESR 910 helium flow cryostat and an ITC503 temperature controller. The experimental conditions were, if not indicated otherwise: 1 mW microwave power, microwave frequency: 9.3-9.4 GHz, 2 G modulation amplitude, 100 kHz modulation frequency. Simulations of the spectra were conducted using the MATLAB toolbox EasySpin (version 5.2.36) with the fit function “pepper”.[5] The following expression was used to fit the power saturation data:

$$\frac{S}{\sqrt{P}} = \frac{1}{\left(1 + \frac{P}{P_{1/2}}\right)^{\frac{b}{2}}}$$

In this expression, *S* denotes the integrated EPR signal intensity, *P* is the applied microwave power, and *b* is a parameter that reflects how homogeneously broadened the spectral line is. Its value ranges from 1 (corresponding to purely inhomogeneous broadening) up to 3 (the limit for homogeneous broadening). A value of $b < 1$ suggests the presence of dipolar interactions.[6-7]

A4. Synthesis and Characterization

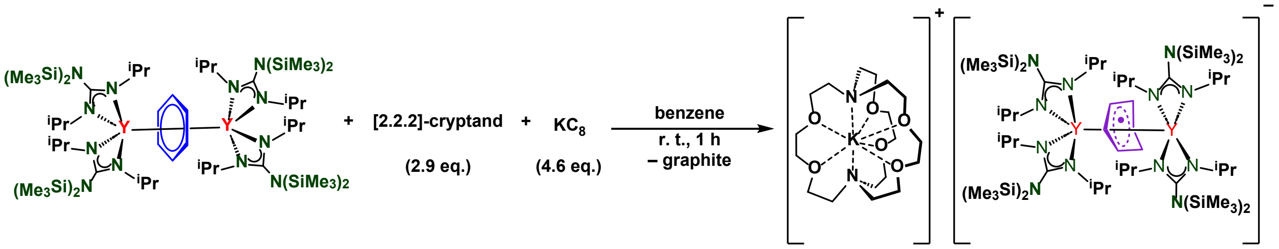

Scheme S1. Synthesis of **2**

Synthesis of [K([2.2.2]-cryptand)][[{(Me$_3$Si)$_2$NC(N^{i}Pr)$_2$}$_2$Y]$_2$(μ–η^6:η^6–C$_6$H$_6^{\bullet}$)] (2): In an argon-filled glovebox, complex **1** (103 mg, 0.0734 mmol, 1.0 equiv) and [2.2.2]-cryptand (79.7 mg, 0.212 mmol, 2.9 equiv) were dissolved in benzene (15 mL) in a 20 mL vial, and a suspension of KC_8 (45.9 mg, 0.339 mmol, 4.6 equiv) in benzene (4 mL) was added at once to the orange-yellow solution. An immediate color change to deep orange-brown was observed. After 1 h of stirring, the reaction mixture was filtered through a glass-microfiber filter and the volatiles of the resulting deep orange-brown solution were removed under vacuum. 3 mL of *n*-hexane was added to the orange-brown residue. After standing at room temperature overnight, the product precipitated as orange-brown crystalline solids, which were suitable for X-ray diffraction analysis. The crystalline solids were collected, washed with *n*-hexane (5×3 mL), and dried under high vacuum to remove the lattice *n*-hexane in the crystals to afford 115.1 mg of the product **2** (86% yield).
Additional information: The reaction with 1 equivalent of [2.2.2]-cryptand works as well. The use of [2.2.2]-cryptand in excess as described above benefits crystal growth in *n*-hexane, suitable for X-ray diffraction analysis.

Elemental analysis calcd for $C_{76}H_{170}KN_{14}O_6Si_8Y_2$: C 50.21, H 9.43, N 10.79. Found: C 50.74, H 9.73, N 10.37.
IR: (ATR, cm^{-1}) 2957m, 2890m, 2820w, 1485s, 1369m, 1361m, 1354m, 1321m, 1311m, 1248s, 1187s, 1165w, 1133m, 1105s, 1078m, 1030s, 951s, 936s, 832s, 753m, 686m, 657m.
UV-vis (1.0·10^{-4} M in Et$_2$O): $\tilde{\nu}$ [10^4 cm^{-1}] / ε [10^4 M^{-1} cm^{-1}] = 3.73 / 2.04, 2.99 / 0.39, 2.37 / 0.44.

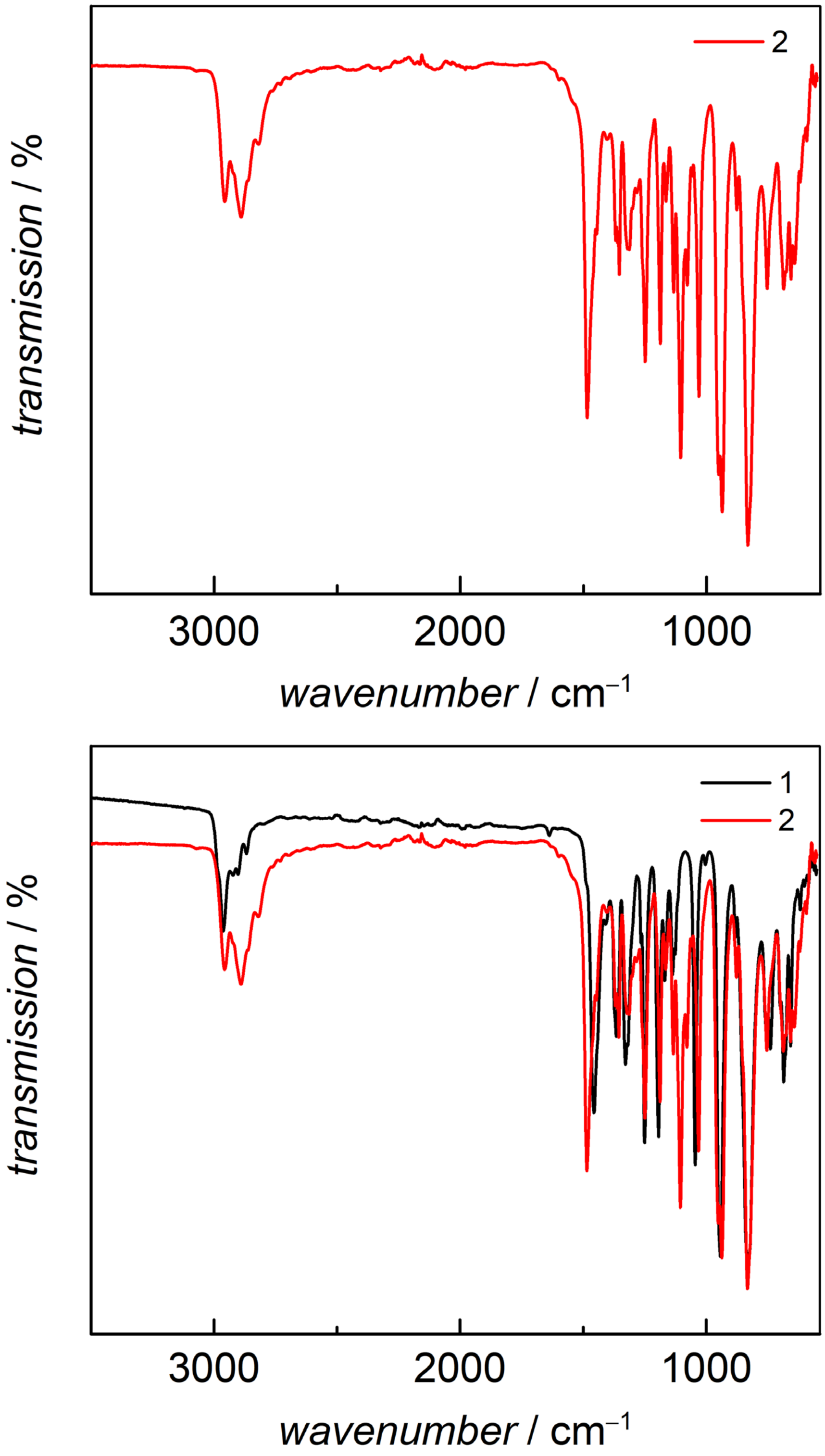

Figure S1. IR vibrational spectrum of [K([2.2.2]-cryptand)][[{$(Me_3Si)_2NC(N^iPr)_2$}$_2Y]_2(\mu\text{–}\eta^6\text{:}\eta^6\text{–}C_6H_6^\bullet)$] (**2**) (upper panel); IR vibrational spectra of [[{$(Me_3Si)_2NC(N^iPr)_2$}$_2Y]_2(\mu\text{–}\eta^6\text{:}\eta^6\text{–}C_6H_6)$] (**1**, black trace) and [K([2.2.2]-cryptand)][[{$(Me_3Si)_2NC(N^iPr)_2$}$_2Y]_2(\mu\text{–}\eta^6\text{:}\eta^6\text{–}C_6H_6^\bullet)$] (**2,** red trace) (lower panel). An IR spectrum of **1** was reported,[1] but **1** was measured here on the same instrument for better comparison to **2**.

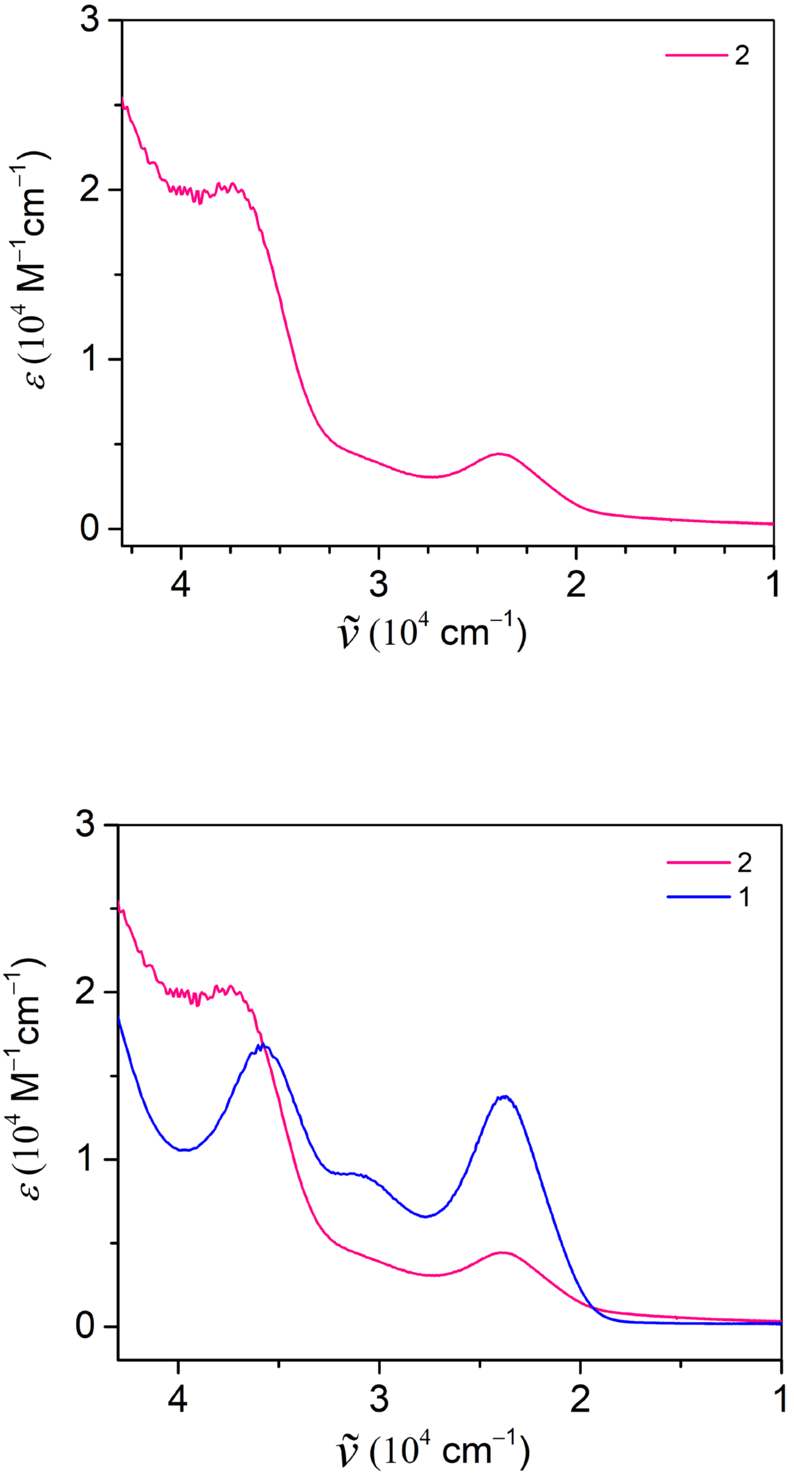

Figure S2. UV-vis electronic absorption spectrum of [K([2.2.2]-cryptand)][[{($Me_3Si)_2NC(N^iPr)_2\}_2Y]_2(\mu\text{–}\eta^6\text{:}\eta^6\text{–}C_6H_6^{\bullet})$] (**2**), recorded at room temperature in Et_2O (upper panel); UV-Vis electronic absorption spectra of [K([2.2.2]-cryptand)][[{($Me_3Si)_2NC(N^iPr)_2\}_2Y]_2(\mu\text{–}\eta^6\text{:}\eta^6\text{–}C_6H_6^{\bullet})$] (**2**, pink trace) and [[{($Me_3Si)_2NC(N^iPr)_2\}_2Y]_2(\mu\text{–}\eta^6\text{:}\eta^6\text{–}C_6H_6)$] (**1**, blue trace), recorded at room temperature in Et_2O (lower panel). The UV-vis spectrum of **1** was reported in hexane,[1] and for better comparison to **2** was measured here on the same instrument in Et_2O.

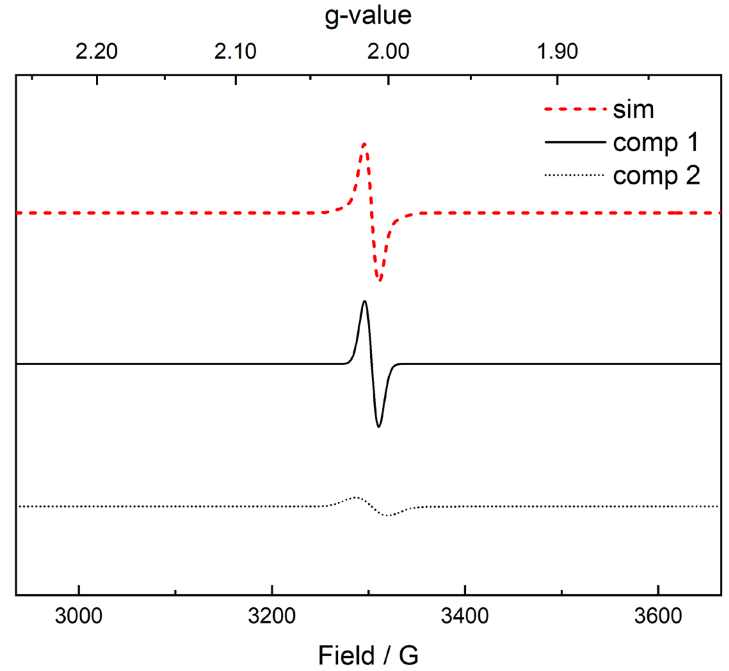

Figure S3. Experimental EPR spectrum of compound **2** in benzene measured at 293 K (Fig. 2a, black trace) and its numerical simulation (red dash trace). The spectra could be best described by two components with different line widths (14 G and 35 G) and equal contribution. These components are tentatively attributed to slightly different conformations of complex **2** in solution. The anion of **2** exhibits dynamic structural flexibility, whereby one bis(guanidinate) yttrium scaffold can rotate relative to the other, causing subtle variations in the electronic structure of the bridging benzene radical trianion ligand and, consequently, in the EPR parameters such as linewidth.

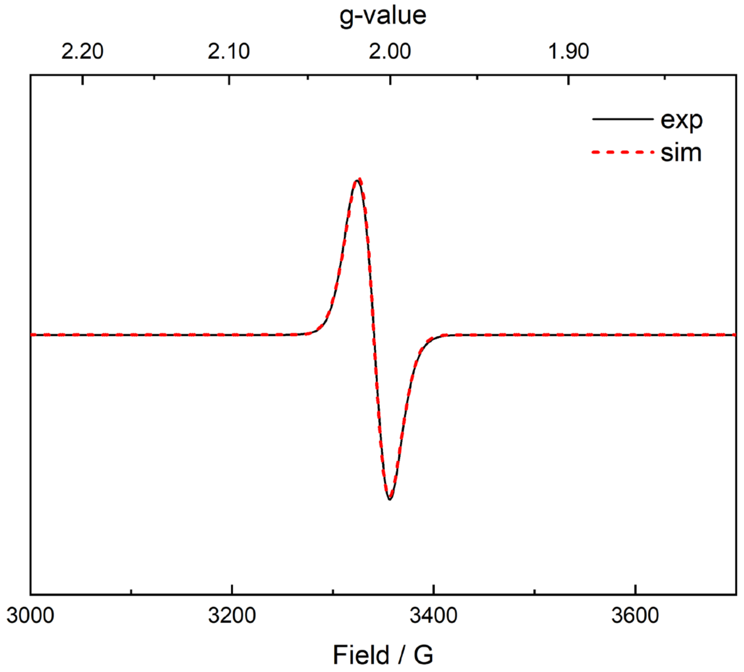

Figure S4. CW X-band EPR spectrum of compound **2**, recorded on crystalline solids, at 80 K (black trace) and its simulation (red dashed trace). Experimental conditions: microwave frequency ν = 9.4 GHz, modulation amplitude = 2 G, microwave power = 1.0 mW, modulation frequency = 100 kHz. Simulation parameters: *g*-value $g = 2.009$, linewidth $W_{FWHM} = 33$ G.

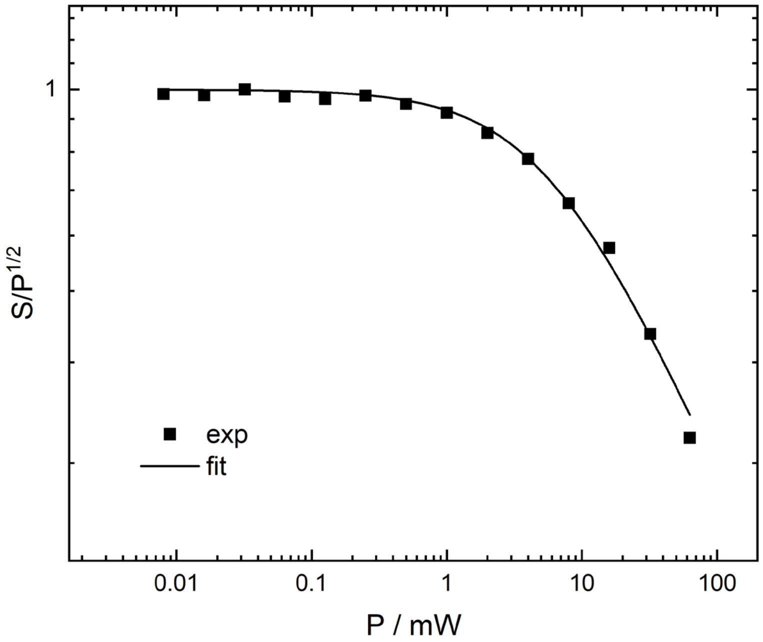

Figure S5. Power saturation curve of compound **2** in benzene recorded at 80 K (black squares) and the corresponding fit (black trace) based on the numerical expression from Portis and Castner.[7-9] The power of half saturation ($P_{1/2}$) is 5.8 mW and *b* = 1.06, which suggests mostly inhomogeneous line broadening and the absence of dipolar coupling to a nearby electron spin.

Table S1. Simulation parameters of the EPR spectra of compound **2**.

Temperature [K]	Phase	g-value	g-strain	H-strain [MHz]	Gaussian line broadening [G]
80 K	crystalline solid	2.0112<br>2.0087<br>2.0083	0.028<br>0.015<br>0.027	20<br>20<br>20	0.5
	Frozen solution	2.0240<br>2.0099<br>1.9980	0.012<br>0.012<br>0.009	20<br>20<br>20	0.5
293 K<br>component 1 (57%)<br>component 2 (43%)	Solution	 2.0105<br>2.0105	 0.01<br>0.023	 10<br>10	 0.3<br>0.3

A5. Details of the Single-Crystal X-ray diffraction analysis

Table S2. Crystal data and structure refinement for **2** • C_6H_{14}.

Empirical formula	C_{82} H_{184} K N_{14} O_6 Si_8 Y_2
Formula weight	1904.06
Temperature	150.15 K
Wavelength	1.54184 Å
Crystal system	Monoclinic
Space group	*C*2/*c*
Unit cell dimensions	a = 23.4513(2) Å, α = 90°
	b = 24.6646(2) Å, β = 98.2256(9)°
	c = 19.2685(2) Å, γ = 90°
Volume	11030.53(18) $Å^3$
Z	4
Density (calculated)	1.147 Mg/m^3
Absorption coefficient	2.955 mm^{-1}
F(000)	4124
Crystal size	0.380 × 0.130 × 0.100 mm^3
Theta range for data collection	2.614 to 72.561°.
Index ranges	-28<=*h*<=21, -30<=*k*<=29, -23<=*l*<=23
Reflections collected	38986
Independent reflections	10814 [*R*(int) = 0.0331]
Completeness to theta = 67.684°	100%
Absorption correction	Semi-empirical from equivalents
Max. and min. transmission	1.00000 and 0.30996
Refinement method	Full-matrix least-squares on F^2
Data/restraints/parameters	10814/0/504
Goodness-of-fit on F^2	1.021
Final *R* indices [*I*>2sigma(*I*)]	R_1 = 0.0309, wR_2 = 0.0765
R indices (all data)	R_1 = 0.0396, wR_2 = 0.0831
Extinction coefficient	n/a
Largest diff. peak and hole	0.348 and -0.493 e. $Å^{-3}$

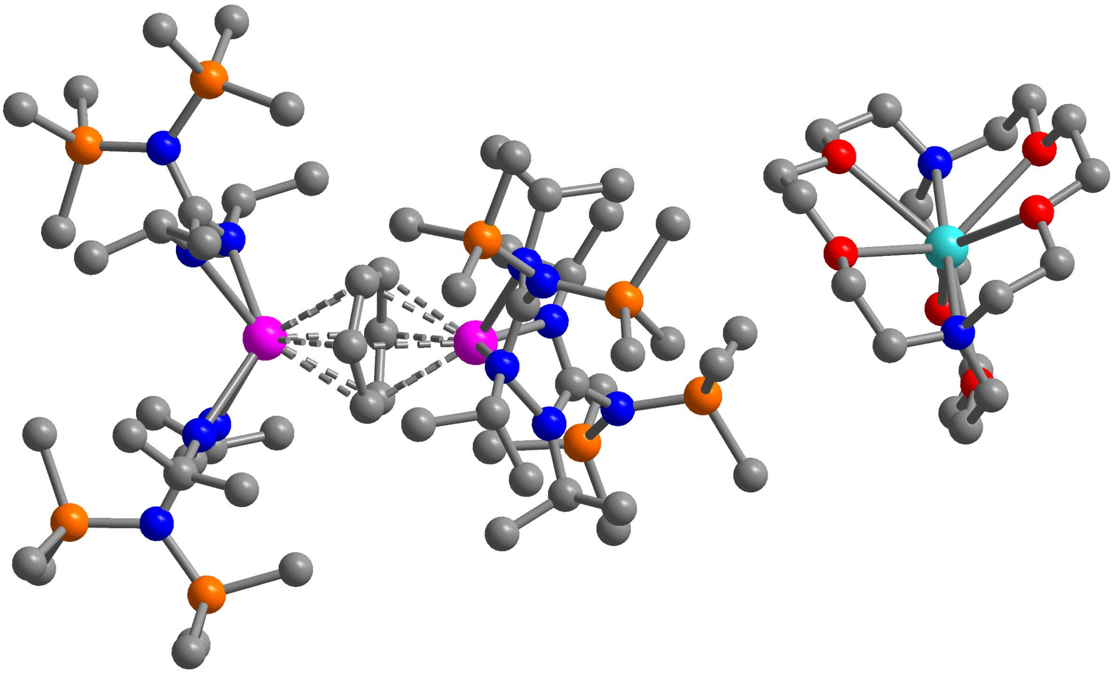

Figure S6. Molecular structure of complex **2**. Pink, orange, blue, red, teal, and gray spheres represent Y, Si, N, O, K, and C atoms, respectively. H atoms are omitted for clarity.

Table S3. Selected distances (Å) and C-C-C-C torsion angles (°) of the benzene dianion in **1**,[1] the benzene radical trianion in **2**, and the benzene radical monoanion in [K(18-crown-6)(η^2-C_6H_6)$_2$][(Cp^{tt}_2La)$_2$(μ-η^6:η^6-$C_6H_6^{\bullet}$)].[10]

	**1**	**2**	[(Cp^{tt}_2La)$_2$(μ-η^6:η^6-$C_6H_6^{\bullet}$)]$^-$
d (C–C)	1.372(3)	1.434(2)	1.450(9)
	1.451(1)	1.434(2)	1.455(8)
	1.490(3)	1.482(3)	1.418(8)
	1.372(3)	1.433(2)	1.450(9)
	1.451(1)	1.433(2)	1.455(8)
	1.490(3)	1.482(3)	1.418(8)
av *d* (C–C)	1.438(2)	1.450(2)	1.441(8)
d (Y1---Y2)	4.515(1)	4.324(1)	
d (Y–C_{arene})	2.662(2)–2.702(2)	2.522(2)–2.681(2)	
av *d* (Y–C_{cent})	2.268(4)	2.162(5)	
av *d* (Y–N_{guan})	2.384(2)	2.458(2)	
C–C–C–C	0.2	16.6	1.4

B. Computational Section

Crystal coordinates of **2** were optimized using the uTPSSh functional[11-12] at def2-TZVP level.[13] To confirm that the optimization resulted in an energetic minimum, frequency calculations were conducted on the optimized coordinates, ensuring the absence of imaginary frequencies. Predicted frequencies were energetically shifted by 124.7 cm^{-1} to better agree with experimentally observed vibrations. Time dependent DFT (TD-DFT) calculations were carried out on the minimized geometry using a manually defined CPCM[14] diethyl ether (Et_2O) solvent model with 250 roots. All calculations were performed using ORCA 5.0.4[15-16] and employed autoaux[17] feature to generate auxiliary basis sets along with the resolution of identity (RI) approximation for the Coulomb integrals, while the exchange integrals were treated with the chain-of-spheres approximation (COSX).[18] Grimme's dispersion correction with Becke-Johnson dampening (D3BJ) was used for all calculations.[19-20] Spin density and molecular orbitals were generated with the orca_plot module and visualizations employed the VMD software.[21] Multiwfn[22] was utilized for the calculation of HOMA and HOMAc values, and mLOBA calculations.[23]

B1. TD-DFT Calculation of 2

Table S4. Majority contributions of the TD-DFT–calculated transition states for **2**, at the def2-TZVP level using the uB3LYP functional with D3BJ dispersion correction and Et_2O implicit solvent model. Isovalue for all depictions is 0.03. Oscillator strength cutoff used is 0.04 and individual contributions higher than 15% are shown. (HOMO = 349, SOMO = 350, LUMO = 351)

<table>
<tr><th>λ (nm)</th><th>ν (cm^{-1})</th><th>Oscillator Strength</th><th>Occupied</th><th>Virtual</th><th>Weight (%)</th></tr>
<tr><td rowspan="3">399.0</td><td rowspan="3">25062</td><td rowspan="3">0.19427</td><td>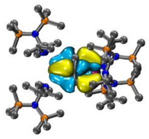
349β</td><td>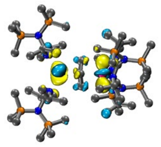
361β</td><td>33.8</td></tr>
<tr><td>349α</td><td>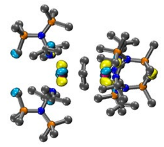
360α</td><td>28.7</td></tr>
<tr><td>349α</td><td>361α</td><td>18.8</td></tr>
<tr><td rowspan="2">269.6</td><td rowspan="2">37091</td><td rowspan="2">0.11411</td><td>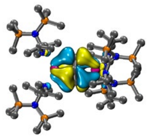
350α</td><td>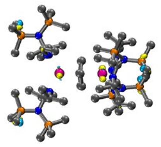
422α</td><td>17.4</td></tr>
<tr><td>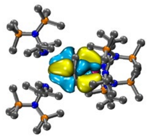
349β</td><td>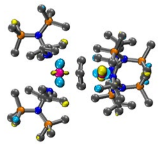
410β</td><td>15.2</td></tr>
</table>

259.7	38511	0.07371	349α	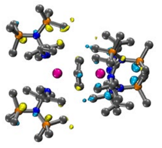 412α	26.6
			350α	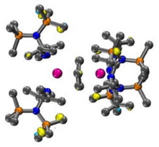 426α	22.2
			349α	410α	18.5
266.5	39156	0.06919	349β	410β	34.9
			349β	412β	26.2
255.4	39156	0.04107	349β	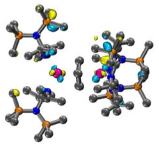 416β	48.9
265.1	37721	0.04089	349α	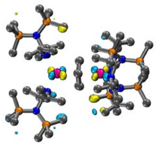 413α	16.9
376.9	26534	0.04032	349β	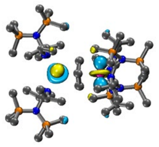 366β	52.6

B2. Bond Metrics and Natural Charges on the Bridging Benzene of 2

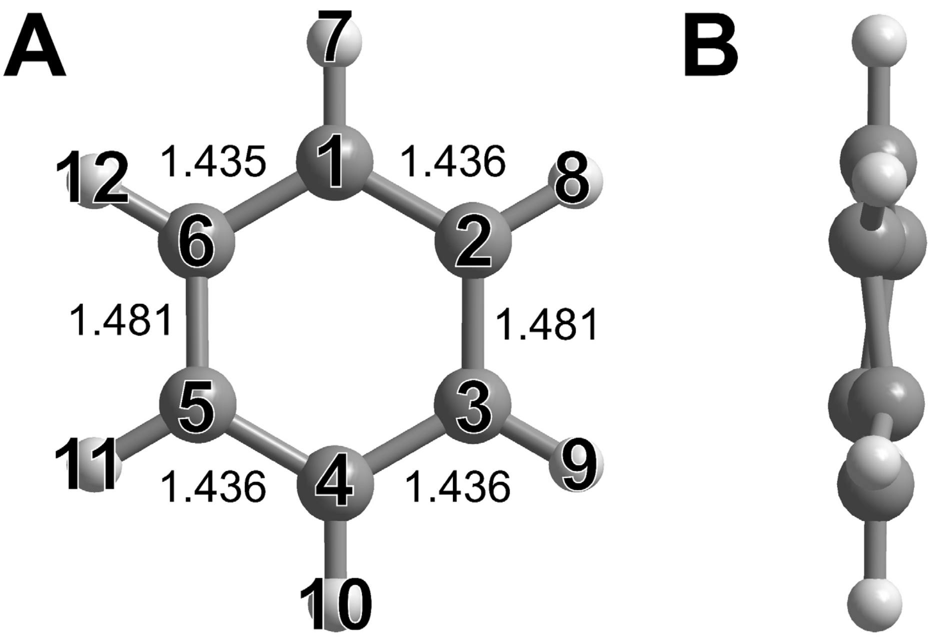

Figure S7. (**A**) Structure of the bridging benzene moiety. The numbers on the atoms represent atomic indices and the numbers between the atoms correspond to C–C bond distances. All bond distances are given in angstrom. (**B**) Side view of the bridging benzene unit depicting the distortion of the arene ring. Gray and white-gray spheres represent C and H atoms, respectively.

Table S5. NPA analysis was employed to calculate natural charges and spin density for the bridging benzene unit of **2**. Oxidation states were calculated by mLOBA analysis.

<table>
<tr><td>Atom</td><td colspan="2">Natural Charge</td><td>Natural Spin Density</td><td colspan="2">Oxidation State</td></tr>
<tr><td>C(1)</td><td>–0.454</td><td rowspan="12">Total charge
= – 1.772</td><td>0.326</td><td>–3</td><td rowspan="12">Total oxidation number
= – 3</td></tr>
<tr><td>C(2)</td><td>–0.585</td><td>0.046</td><td>–1</td></tr>
<tr><td>C(3)</td><td>–0.585</td><td>0.046</td><td>–1</td></tr>
<tr><td>C(4)</td><td>–0.454</td><td>0.327</td><td>–1</td></tr>
<tr><td>C(5)</td><td>–0.585</td><td>0.046</td><td>–3</td></tr>
<tr><td>C(6)</td><td>–0.585</td><td>0.046</td><td>0</td></tr>
<tr><td>H(7)</td><td>0.248</td><td>–0.011</td><td>1</td></tr>
<tr><td>H(8)</td><td>0.245</td><td>–0.001</td><td>1</td></tr>
<tr><td>H(9)</td><td>0.245</td><td>–0.001</td><td>1</td></tr>
<tr><td>H(10)</td><td>0.248</td><td>–0.011</td><td>1</td></tr>
<tr><td>H(11)</td><td>0.245</td><td>–0.001</td><td>1</td></tr>
<tr><td>H(12)</td><td>0.245</td><td>–0.001</td><td>1</td></tr>
<tr><td>Y</td><td colspan="2">1.788</td><td>0.068</td><td colspan="2">3</td></tr>
<tr><td>Y</td><td colspan="2">1.788</td><td>0.068</td><td colspan="2">3</td></tr>
</table>

B3. Harmonic Oscillator Model of Aromaticity (HOMA) and HOMAc Values of 2

Table S6. HOMA values calculated for the bridging benzene unit of **2**.

Atom Pair	Contribution	Bond Distance (Å)
1(C) – 2(C)	–0.096650	1.435437
2(C) – 3(C)	–0.373839	1.481295
3(C) – 4(C)	–0.097255	1.435586
4(C) – 5(C)	–0.096911	1.435501
5(C) – 6(C)	–0.373775	1.481288
6(C) – 1(C)	–0.096949	1.435511

Final HOMA value = –0.135379

Table S7. HOMAc values calculated for the bridging benzene unit of **2**.

Atom Pair	Contribution	Bond Distance (Å)
1(C) – 2(C)	–0.048230	1.435437
2(C) – 3(C)	–0.203821	1.481295
3(C) – 4(C)	–0.048559	1.435586
4(C) – 5(C)	–0.048372	1.435501
5(C) – 6(C)	–0.203784	1.481288
6(C) – 1(C)	–0.048393	1.435511

Final HOMAc value = 0. 398841

B4. Geometry optimized coordinates of 2. Optimization was conducted using uTPSSh functional at def2-TZVP level. (final energy = -5343.266170327661 Hartrees)

C	-0.687986	9.895548	4.767640
H	-0.687989	10.974892	4.767546
C	-1.194443	9.181832	5.905441
C	-0.181518	9.181619	3.629885
Y	1.336731	8.447684	5.498202
Y	-2.712878	8.447770	4.037394
H	-1.406453	9.720922	6.814728
C	-1.023086	7.711785	5.967367
H	-1.438153	7.173162	6.804118
C	-0.688199	6.997871	4.767749
C	-0.353116	7.711599	3.568176
H	-0.688309	5.918515	4.767800
C	3.536569	7.694931	2.508148
H	4.193720	6.845051	2.293172
C	4.364656	8.970515	2.350115
C	2.411036	7.704426	1.470873
N	3.001288	7.562930	3.851787
H	3.749660	9.850745	2.545782
H	4.755959	9.046162	1.330366
H	5.208632	8.984370	3.041107
H	1.831053	6.780752	1.508794
H	2.814453	7.818296	0.459067
H	1.733065	8.535658	1.670945
C	2.713728	5.265892	6.630891
H	3.128009	4.412070	6.079608
C	3.644194	5.564500	7.811540
C	1.319345	4.884967	7.133739
N	2.610539	6.416913	5.756037
H	3.293025	6.449975	8.339776
H	4.660219	5.763994	7.468687
H	3.675891	4.725149	8.514876
H	1.371607	4.062257	7.854125
H	0.675526	4.586006	6.305685
H	0.845236	5.744441	7.614374
C	1.463056	4.261741	3.348257
H	1.144620	3.849735	4.308204
H	0.950549	3.708604	2.555205
H	1.126777	5.300784	3.312479
Si	3.317563	4.144888	3.141325
C	3.745110	4.269491	1.309727
H	3.338898	5.172928	0.851241
H	3.323250	3.408386	0.778907
H	4.825157	4.264859	1.141501
C	3.918013	2.448808	3.701500
H	4.973963	2.284016	3.468410
H	3.345872	1.671274	3.183749
H	3.782349	2.307269	4.776862
C	6.829744	5.339800	2.980493
H	6.738657	4.355285	2.512068
H	7.884907	5.481348	3.238918
H	6.561257	6.093824	2.235700
Si	5.761256	5.487599	4.526329

C	6.304756	4.093528	5.673849
H	5.667979	4.014340	6.556966
H	7.330029	4.276066	6.014797
H	6.294425	3.125424	5.167334
C	6.098120	7.139720	5.345929
H	6.104615	7.949525	4.614513
H	7.075619	7.121056	5.838597
H	5.343245	7.384919	6.095161
C	3.113086	11.631919	5.505519
H	3.075051	12.487368	6.192050
C	4.585276	11.337183	5.197780
C	2.364740	12.006052	4.224100
N	2.475038	10.480931	6.112791
H	4.657962	10.450213	4.569684
H	5.144972	11.142353	6.113368
H	5.058252	12.176752	4.676637
H	2.866381	12.825589	3.699531
H	1.339837	12.306338	4.445872
H	2.308409	11.142148	3.556984
C	1.117069	9.200820	9.204094
H	1.489257	10.048224	9.790481
C	-0.411849	9.200600	9.281917
C	1.643166	7.920981	9.854207
N	1.566724	9.331181	7.829146
H	-0.828345	10.125165	8.878655
H	-0.749099	9.092311	10.318260
H	-0.810473	8.369674	8.697728
H	1.293786	7.044609	9.305376
H	1.285460	7.843720	10.886085
H	2.733759	7.901036	9.869550
C	2.191128	10.420127	7.409954
N	2.551408	11.500861	8.308214
C	4.896281	9.745981	8.655002
H	4.817363	9.517465	7.590719
H	4.413391	8.931619	9.197430
H	5.956980	9.751675	8.926098
Si	4.117299	11.397598	9.078262
C	5.272202	12.792968	8.554629
H	6.279318	12.603448	8.942552
H	4.944515	13.757935	8.948824
H	5.343308	12.883202	7.469051
C	3.946931	11.532636	10.950607
H	3.579193	12.515903	11.258718
H	4.922165	11.385384	11.427499
H	3.261712	10.777506	11.345027
C	2.181818	14.448172	8.529530
H	2.842342	14.613441	9.385621
H	1.411671	15.226654	8.558470
H	2.767916	14.587996	7.617533
Si	1.359035	12.753431	8.576404
C	0.516997	12.630174	10.258323
H	-0.089811	11.727640	10.351284
H	-0.145322	13.492289	10.397295
H	1.240265	12.633391	11.077865

C	0.064075	12.639889	7.232473
H	0.436535	13.042348	6.288024
H	-0.831663	13.203296	7.511443
H	-0.227250	11.602371	7.051854
C	3.216782	6.475587	4.574569
N	4.065921	5.394148	4.112017
H	0.030377	9.720554	2.720471
C	-3.567409	10.419042	2.124379
C	-4.594894	6.477831	4.961848
N	-3.989128	6.418754	3.780145
N	-2.942948	9.329979	1.705547
N	-3.851464	10.480181	3.421484
N	-4.378499	7.565092	5.684460
H	0.061706	7.172807	2.731422
N	-3.928622	11.499118	1.225666
N	-5.444729	5.397037	5.424650
C	-4.093442	5.267975	2.905125
C	-2.492657	9.199550	0.330801
C	-4.490126	11.631001	4.028379
C	-4.913077	7.697339	7.028365
Si	-5.494672	11.394415	0.456137
Si	-2.737182	12.752350	0.956427
Si	-7.140110	5.491743	5.010813
Si	-4.697079	4.147281	6.395250
H	-4.508430	4.414419	3.456288
C	-5.023722	5.567809	1.724647
C	-2.699418	4.885925	2.402106
H	-2.863878	10.047371	-0.255609
C	-0.963688	9.198162	0.253866
C	-3.019392	7.920302	-0.319962
H	-4.452413	12.486317	3.341659
C	-5.962191	11.335465	4.335926
C	-3.742126	12.005760	5.309821
H	-5.569875	6.847336	7.243958
C	-5.741512	8.972683	7.186576
C	-3.786904	7.707458	8.064935
C	-6.272782	9.742883	0.881252
C	-6.650114	12.789691	0.978831
C	-5.324871	11.527784	-1.416377
C	-3.561594	14.446388	1.000029
C	-1.893811	12.627252	-0.724693
C	-1.443131	12.642159	2.301504
C	-8.208129	5.345139	6.557078
C	-7.685086	4.097902	3.863716
C	-7.475883	7.143900	4.190864
C	-2.842576	4.261892	6.186998
C	-5.123129	4.273048	8.227123
C	-5.299958	2.451725	5.836109
H	-4.671905	6.453329	1.196928
H	-6.039588	5.767880	2.067634
H	-5.056057	4.728864	1.020856
H	-2.752421	4.063465	1.681490
H	-2.055806	4.586188	3.230048
H	-2.224587	5.745119	1.921688

H	-0.546681	10.122100	0.658037
H	-0.625938	9.090368	-0.782363
H	-0.566038	8.366487	0.837653
H	-2.671382	7.043476	0.229006
H	-2.660831	7.842900	-1.351532
H	-4.109985	7.901403	-0.336331
H	-6.034385	10.448402	4.963938
H	-6.521659	11.140338	3.420252
H	-6.435734	12.174726	4.857051
H	-4.244248	12.825120	5.834203
H	-2.717349	12.306538	5.088117
H	-3.685420	11.141990	5.977076
H	-5.127080	9.853122	6.990109
H	-6.132036	9.048505	8.206611
H	-6.586059	8.985940	6.496276
H	-3.206570	6.784013	8.026778
H	-4.189743	7.821300	9.076971
H	-3.109386	8.538939	7.864343
H	-6.193915	9.515775	1.945842
H	-5.789342	8.928093	0.339961
H	-7.333440	9.747644	0.609972
H	-7.657164	12.599491	0.591066
H	-6.322841	13.754539	0.584000
H	-6.721230	12.880601	2.064354
H	-4.957981	12.511045	-1.725508
H	-6.300088	11.379298	-1.892920
H	-4.639129	10.772825	-1.810216
H	-4.221621	14.609664	0.143171
H	-2.792174	15.225565	0.970445
H	-4.148529	14.587087	1.911352
H	-1.286629	11.724805	-0.816035
H	-1.231674	13.489409	-0.864252
H	-2.616445	12.629184	-1.544791
H	-1.816385	13.046545	3.244810
H	-0.547351	13.205215	2.021958
H	-1.151652	11.605131	2.484605
H	-8.117529	4.360704	7.025769
H	-9.263299	5.487355	6.299056
H	-7.938783	6.099205	7.301522
H	-7.048762	4.018229	2.980321
H	-8.710388	4.281104	3.523208
H	-7.675171	3.129831	4.370299
H	-7.481289	7.954001	4.921963
H	-8.453618	7.125956	3.698637
H	-6.721120	7.388100	3.441194
H	-2.525163	3.848360	5.227369
H	-2.330178	3.709183	6.980418
H	-2.505145	5.300615	6.221287
H	-4.715401	5.176122	8.684981
H	-4.701960	3.411591	8.757919
H	-6.203052	4.269858	8.396176
H	-6.355938	2.288294	6.070027
H	-4.728380	1.673673	6.353703
H	-5.165258	2.309666	4.760694

B5. Calculations on Hypothetical Molecules

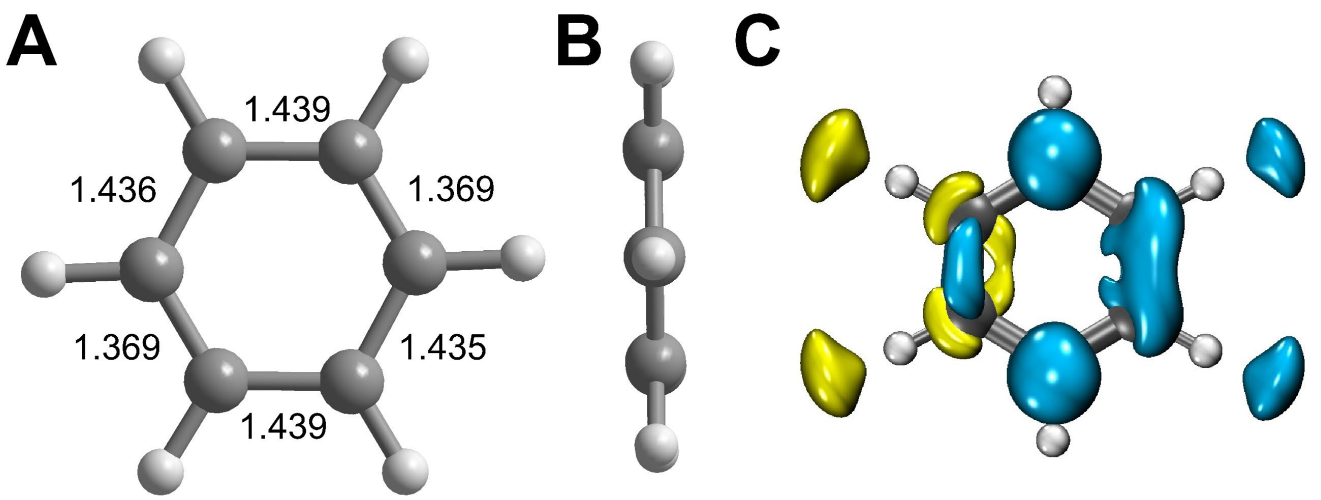

Figure S8. (**A**) Front view displaying bond distances in angstrom, (**B**) side view, and (**C**) spin density calculated for the bare benzene trianion. Gray and white-gray spheres represent C and H atoms. Isovalue for spin density surfaces set to 0.005.

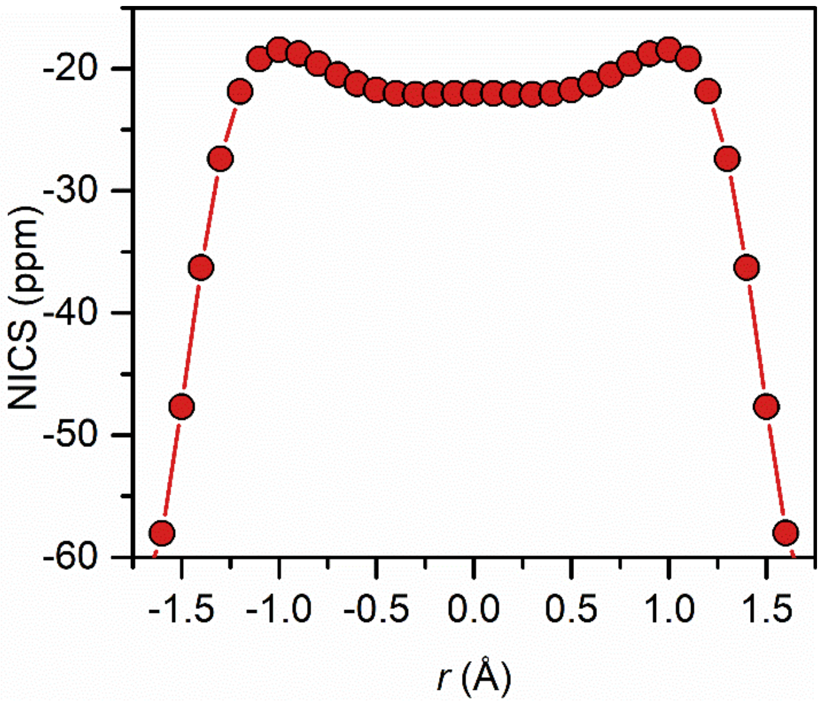

Figure S9. Calculated NICS values for the hypothetical complex $[[\{(Me_3Si)_2NC(N^iPr)_2\}_2Y]_2(\mu\text{–}\eta^6\text{:}\eta^6\text{–}C_6H_6)]^{2-}$ comprising a bridging benzene tetraanion.

C. References

[1] F. Delano IV, S. Demir, *Angew. Chem. Int. Ed.* **2025**, *64*, e202417217.
[2] G. M. Sheldrick, *Acta Crystallogr. Sect. C Struct. Chem.* **2015**, *71*, 3–8.
[3] L. J. Bourhis, O. V. Dolomanov, R. J. Gildea, J. A. Howard, H. Puschmann, *Acta Crystallogr. A: Found. Adv.* **2015**, *71*, 59–75.
[4] O. V. Dolomanov, L. J. Bourhis, R. J. Gildea, J. A. K. Howard, H. Puschmann, *J. Appl. Crystallogr.* **2009**, *42*, 339–341.
[5] S. Stoll, A. Schweiger, *J. Magn. Reson.* **2006**, *178*, 42–55.
[6] H. Rupp, K. K. Rao, D. O. Hall, R. Cammack, *Biochim. Biophys. Acta* **1978**, *537*, 255–269.
[7] D. J. Hirsh, G. W. Brudvig, *Nat. Protoc.* **2007**, *2*, 1770–1781.
[8] T. G. Castner, *Phys. Rev.* **1959**, *115*, 1506–1515.
[9] A. M. Portis, *Phys. Rev.* **1953**, *91*, 1071–1078.
[10] M. C. Cassani, D. J. Duncalf, M. F. Lappert, *J. Am. Chem. Soc.* **1998**, *120* 12958−12959.
[11] V. N. Staroverov, G. E. Scuseria, J. Tao, J. P. Perdew, *J. Chem. Phys.* **2003**, *119*, 12129–12137.
[12] J. Tao, J. P. Perdew, V. N. Staroverov, G. E. Scuseria, *Phys. Rev. Lett.* **2003**, *91*, 146401.
[13] F. Weigend, R. Ahlrichs, *Phys. Chem. Chem. Phys.* **2005**, *7*, 3297–3305.
[14] V. Barone, M. Cossi, *J. Phys. Chem. A* **1998**, *102*, 1995–2001.
[15] F. Neese, *WIREs Comput. Mol. Sci.* **2011**, *2*, 73–78.
[16] F. Neese, *WIREs Comput. Mol. Sci.* **2022**, *12*, e1606.
[17] G. L. Stoychev, A. A. Auer, F. Neese, *J. Chem. Theory. Comput.* **2017**, *13*, 554–562.
[18] F. Neese, F. Wennmohs, A. Hansen, U. Becker, *Chem. Phys.* **2009**, *356*, 98–109.
[19] S. Grimme, J. Antony, S. Ehrlich, H. A. Krieg, *J. Chem. Phys.* **2010**, *132*, 154104.
[20] S. Grimme, S. Ehrlich, L. Goerigk, *J. Comput. Chem.* **2011**, *32*, 1456–1465.
[21] W. Humphrey, A. Dalke, K. Schulten, *J. Mol. Graph.* **1996**, *14*, 33–38.
[22] T. Lu, *J. Chem. Phys.* **2024**, *161*, 082503.
[23] A. J. Thom, E. J. Sundstrom, M. Head-Gordon, *Phys. Chem. Chem. Phys.* **2009**, *11*, 11297–11304.
